# Intussusception With a Pathological Lead Point in a Two-Month-Old Infant

**DOI:** 10.7759/cureus.59273

**Published:** 2024-04-29

**Authors:** Erin M Sanzone, Ashley Moore, Alexis Sieber, Anita S Nathan, Erika Lindholm

**Affiliations:** 1 Department of Pediatrics, Cooper Medical School of Rowan University, Camden, USA; 2 Department of Pediatrics, Cooper University Hospital, Camden, USA; 3 Department of Pediatric Surgery, Cooper University Hospital, Camden, USA

**Keywords:** myoglandular-type polyps, pathological lead point, intussusception

## Abstract

Intussusception is one of the most common causes of acute intestinal obstruction in infancy and early childhood. Most cases of intussusception tend to occur in infancy, between the ages of four and six months. The causes can be split into two categories: non-pathologic and pathologic. Non-pathological causes include administration of the rotavirus vaccine, dehydration, and recent illness. Pathological causes can be attributed to Meckel’s diverticulum (in 75% of cases), polyps (15%), and lymphoma or other tumors (3%). Intussusception rarely occurs in infants less than three months of age. If intussusception does occur in patients under three months of age, the cause is idiopathic in up to 75% of the cases. Additionally, myoglandular-type polyps are exceedingly rare and very rarely occur in patients under the age of 50. This case report discusses the diagnosis and treatment of intussusception in a two-month-old male patient who initially presented to the pediatric inpatient unit for dehydration secondary to a suspected viral illness, later developing colicky abdominal pain and bloody stools. He was found to have colo-colonic intussusception with a myoglandular-type polyp lead point. In discussing this case, the aim is to teach about intussusception and myoglandular-type polyps, as well as reveal a rarity in both etiologies for this age group.

## Introduction

Intussusception is a rare but serious condition in the pediatric population that occurs when part of the intestine invaginates within the next section, possibly causing obstruction, ischemia, and perforation if not immediately treated [1]. Intussusception has an average effect of 30 cases per 100,000 children under three years [2]. It mainly occurs in patients aged six to 18 months. An analysis of the etiology of intussusception shows that the cause is often unknown; however, risk factors for intussusception include infections, cystic fibrosis, and intestinal polyps [1]. Pathological lead points include lymphoid hyperplasia, Meckel’s diverticulum, Burkitt’s lymphoma, and juvenile polyps [3]. The initial treatment for intussusception is reduction with a contrast enema. If reduction fails, the patient undergoes surgical treatment [3]. A few reasons for intussusception reduction failure include the distal mass and observation of the dissecting sign [3].

Nakamura et al. first described inflammatory myoglandular polyps, which share histological features of inflammatory polyps, juvenile polyps, and Peutz-Jeghers polyps [4,5]. Inflammatory myoglandular polyps show proliferation of smooth muscle in the center, extending radially to the lamina propria, inflammatory granulation tissue in the stroma, and hyperplastic glands with occasional cystic dilation [6]. The mean age of inflammatory myoglandular polyps is 49 years [6]. Polyps were found in the rectum, sigmoid colon, descending colon, and distal transverse colon. Inflammatory myoglandular polyps have not been associated with neoplasms to date; therefore, they are considered benign [7].

This case report uncovers an extremely rare clinical scenario in which a two-month-old pediatric patient presented with blood in stool and was found to have colo-colonic intussusception as well as pedunculated myoglandular-type polyps within the lesion. Our goal was to present an analysis of the case presentation, including the patient’s history, challenges faced, working differentials, therapeutic intervention, and outcomes. Although polyps are extremely rare under one year of age and intussusception is extremely rare under six months, physicians must consider the diagnosis of juvenile polyps and intussusception in a wider age range of patients presenting with bloody stools. We emphasize the importance of early diagnosis and intervention to improve patient outcomes in patients with juvenile polyps complicated by intussusception.

## Case presentation

A two-month-old male patient, born full-term via cesarean section with no significant past medical history, was admitted to the hospital for vomiting. He had a week-long history of cough, congestion, runny nose, loose stool with mucus, and four days of non-bilious, non-bloody emesis. Within the past day, the parents also noted three episodes of blood in stool and decreased oral intake and urine output. The parents stated that they had taken him to an outside hospital four days prior with the same symptoms, where he was provided oral hydration and sent home for supportive treatment and return precautions. His mother recently had an upper respiratory tract infection.

The initial ED workup revealed sunken fontanelles and decreased activity. A basic metabolic panel (BMP) portrayed hyperglycemia, hyponatremia, and hypercalcemia with high anion gap acidosis (Table 1). Two hours later, a repeat BMP revealed continued hyperglycemia, an improved sodium level, an improved bicarbonate level, and an improved anion gap. He also had leukocytosis with a normal differential, thrombocytosis, and mild transaminitis. This presentation is likely to be secondary to dehydration. He was negative for COVID-19, influenza, and respiratory syncytial virus.

**Table 1:** BMP at admission and two-hour repeat labs The labs portray hyperglycemia, hyponatremia, and hypercalcemia with high anion gap acidosis. Two hours later, a repeat BMP revealed continued hyperglycemia, an improved sodium level, an improved bicarbonate level, and an improved anion gap. BMP, basic metabolic panel

Component	Result	Repeat the result two hours later	Reference range
Glucose (mg/dL)	147	169	70-100
BUN (mg/dL)	13	12	2-13
Creatinine (mg/dL)	0.28	0.28	0.4-0.7
Sodium (mmol/L)	129	134	134-142
Potassium (mmol/L)	4.5	5	3.5-5.6
Chloride (mmol/L)	96	103	96-110
CO_2 _(mmol/L)	15	16	20-28
Calcium (mg/dL)	10.7	9.6	8.7-10.5
Anion gap (mmol/L)	18	15	7-15

**Figure 1:**
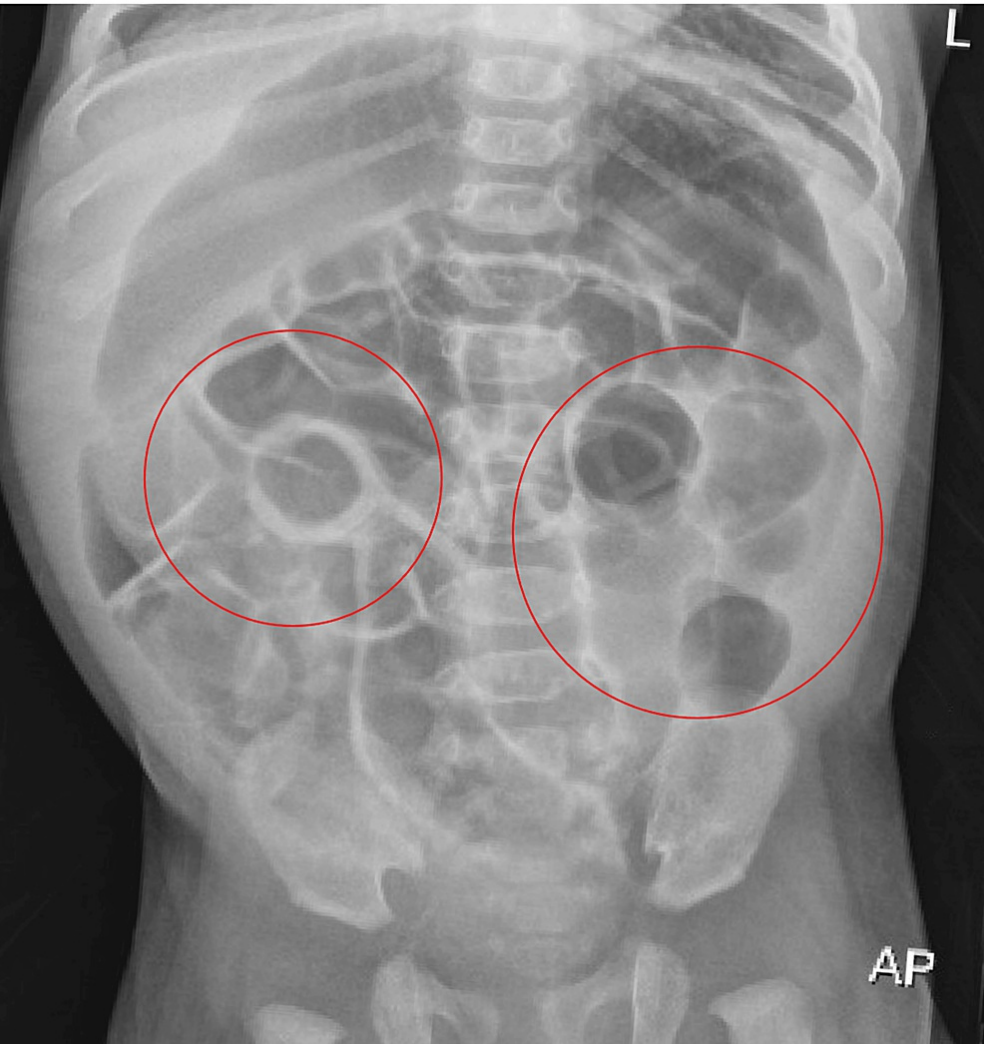
Obstruction series Mildly distended loops of small bowel (circled in red) are visualized throughout the abdomen. There is no evidence of free peritoneal air. Lungs are clear and well expanded. AP, anteroposterior

Pediatric surgery was consulted, and an obstruction series showed dilated small bowel loops (Figure 1) with no immediate intervention needed. The patient received two normal saline (NS) boluses in the ED and two on the floor. However, he had persistent loose stools, tachycardia, and signs of dehydration. The patient was given a third NS bolus at that time, in addition to increasing the maintenance IV fluid rate to 1.5× maintenance. He had one blood pressure of 52/22 mmHg with a repeat soon after 128/67 mmHg. The patient was transferred to the pediatric intensive care unit (PICU) for escalated care. In the PICU, he was stabilized with the maintenance of intravenous fluids.

On hospital day 2, the patient was transferred back to the pediatric step-down unit, and oral feeding was initiated. On hospital day 3, concerns arose regarding left upper extremity swelling, suggesting intravenous fluid infiltration. Orthopedic surgeons evaluated the patient for possible compartment syndrome. However, after an adequate workup, they successfully ruled out compartment syndrome, with recommendations to keep his arm elevated and heating packs as tolerated. Simultaneously, a *Clostridium difficile *infection was identified during the infectious workup.

**Figure 2:**
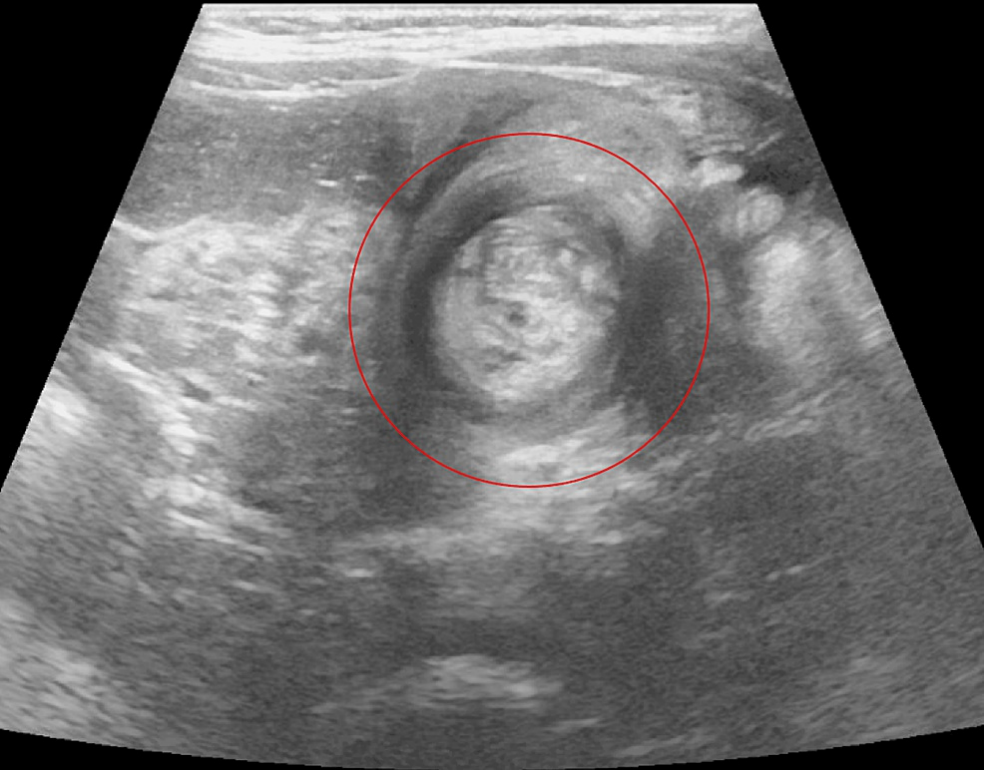
Ultrasound findings of intussusception Intussusception measures at least 3 cm in cross-section, suggesting a colonic component, most likely ileocolic. Findings compatible with intussusception were favored to be ileocolic. The telescoping bowel is circled in red.

**Figure 3:**
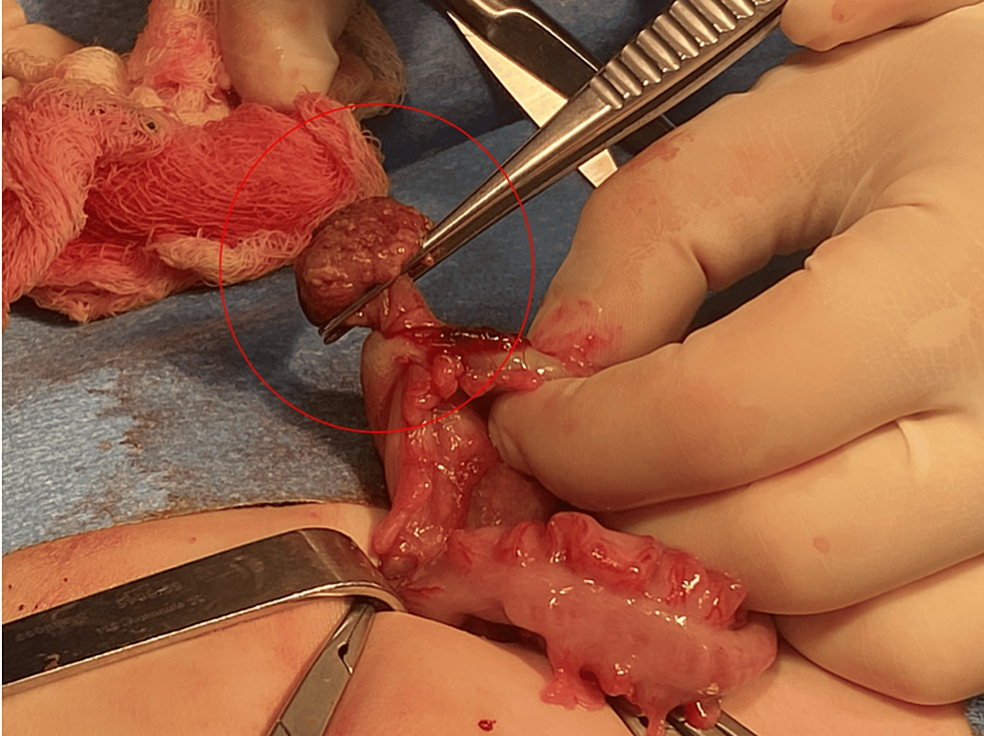
Intraoperative photos of polyp A 2-cm pedunculated polyp was identified (circled in red). The polyp was excised, and there was no additional pathology identified. The mucosa in the surrounding tissue was edematous, but no additional masses were identified.

**Figure 4:**
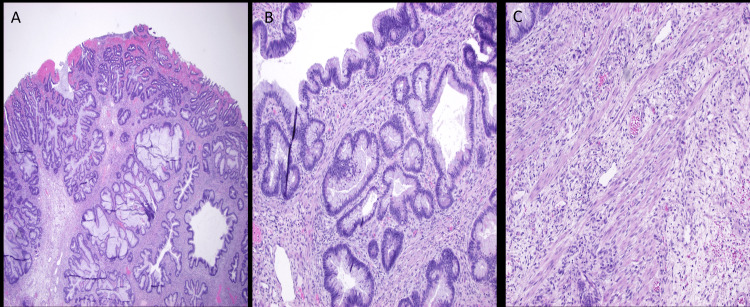
Myoglandular-type polyp A-C (H&E): A 2.1-cm polyp with a focally eroded surface composed of multiple complex glandular structures lined by goblet cell-rich columnar epithelium (seen in A). The cellular stroma shows mild patchy mixed inflammation, somewhat primitive mesenchymal cells, and numerous strands of smooth muscle (seen in B and C) that orient perpendicular and parallel to the surface. Per the outside report, there are overlapping features between an epithelial-rich juvenile polyp and a hamartomatous polyp.

By hospital day 5, his parents had reported an episode of bloody stools. The pediatric team initially attributed hematochezia to a possible milk protein allergy or viral gastroenteritis. The patient switched to the Alimentum formula. Due to the persistence of symptoms of blood in stool and diminished oral intake, a nasoduodenal (ND) tube was inserted, and imaging was recommended. Ultrasonography revealed intussusception, likely ileocolic (Figure 2). The initial reduction of intussusception with an air enema failed. The patient was taken to the OR. There was no sign of intussusception in the cecum. The colon was soft and had no signs of a telescoped bowel. Following the colon, there was an abnormality in the left lower quadrant (LLQ) sigmoid region where a colo-colonic intussusception was identified. The LLQ sigmoid region posed challenges laparoscopically, necessitating an open LLQ incision with colotomy and excision. The intussusception was reduced successfully. There was a hard mass identified within the bowel. The mass was excised. As seen in Figure 3, a 2-cm pedunculated polyp was identified, and an enterotomy revealed a myoglandular-type polyp (Figure 4).

Postoperatively, the patient tolerated the reintroduction of oral feeding with the original Similac-sensitive formula. No occurrences of bloody stool were noted, and there was no continued concern for a milk protein allergy, as no symptoms continued after surgery.

On hospital day 7, he was discharged following a successful postoperative recovery with plans for pediatric surgery follow-up.

## Discussion

Myoglandular polyps are a unique and rare form of polyp, especially in infants. A case series from 2002 studied 1,500 patients with colorectal polyps undergoing endoscopic polypectomy, with 0.6% found to have inflammatory myoglandular polyps and a mean age of 49 years [6].

In 1993, in the UK, a 72-year-old female was found to have an inflammatory myoglandular polyp causing ileo-ileal intussusception [8]. The presenting symptoms were four to five episodes of colicky lower abdominal pain over three to four months, occurring mainly after eating. On a physical exam, she was found to have a tender, soft, mobile mass palpable in her right iliac fossa. The differential diagnosis originally was right-sided colonic carcinoma. However, during the operation, an intussuscepting pedunculated polyp was found at the terminal ileum and histopathologically found to be an inflammatory myoglandular polyp lead point [8].

There is a case report of a nine-year-old female with no significant past medical history or family history presenting with bleeding per rectum over 10 days. The proctoscopy revealed a polyp measuring 1.2 cm with a small peduncle situated 5 cm proximal to the anal verge on the anterior wall of the rectum. Immunohistopathological findings discovered this was an inflammatory myoglandular polyp [4]. This polyp type is exceedingly rare for a nine-year-old; our patient at two months of age is even more rare. Furthermore, the polyp was in the rectal area for the nine-year-old female, as opposed to our patient in the colo-colonic region. Both presented with bloody stools.

Our patient is unique in presentation and age range for both intussusception and myoglandular polyp, underscoring the importance of considering a broad differential diagnosis and maintaining a high index of suspicion for rare conditions. The definitive diagnosis of inflammatory myoglandular polyp in all reported cases was confirmed through histopathological examination, emphasizing the importance of this diagnostic step to guide management and treatment.

In summary, these cases emphasize the variability in age, location, and clinical presentation of inflammatory myoglandular polyps leading to intussusception and underscore the need for a comprehensive and individualized diagnostic approach in pediatric patients presenting with gastrointestinal symptoms to ensure timely diagnosis and appropriate management.

## Conclusions

The presented case details a two-month-old male infant who was admitted to the hospital with symptoms of vomiting, cough, congestion, runny nose, loose stool with mucus, and episodes of bloody stool and was found to have colo-colonic intussusception with a myoglandular-type polyp lead point. This case highlights the importance of considering intussusception and juvenile polyps in the differential diagnosis of pediatric patients presenting with bloody stool, even in very young infants. It also underscores the significance of prompt diagnosis, thorough evaluation, and timely surgical intervention to achieve favorable outcomes in complex pediatric gastrointestinal cases.
